# Quantitative Measurement of Brightness from Living Cells in the Presence of Photodepletion

**DOI:** 10.1371/journal.pone.0097440

**Published:** 2014-05-12

**Authors:** Kwang-Ho Hur, Patrick J. Macdonald, Serkan Berk, C. Isaac Angert, Yan Chen, Joachim D. Mueller

**Affiliations:** 1 School of Physics and Astronomy, University of Minnesota, Minneapolis, Minnesota, United States of America; 2 Department of Biomedical Engineering, University of Minnesota, Minneapolis, Minnesota, United States of America; CNR, Italy

## Abstract

The brightness of fluorescently labeled proteins provides an excellent marker for identifying protein interactions in living cells. Quantitative interpretation of brightness, however, hinges on a detailed understanding of the processes that affect the signal fluctuation of the fluorescent label. Here, we focus on the cumulative influence of photobleaching on brightness measurements in cells. Photobleaching within the finite volume of the cell leads to a depletion of the population of fluorescently labeled proteins with time. The process of photodepletion reduces the fluorescence signal which biases the analysis of brightness data. Our data show that even small reductions in the signal can introduce significant bias into the analysis of the data. We develop a model that quantifies the bias and introduce an analysis method that accurately determines brightness in the presence of photodepletion as verified by experiments with mammalian and yeast cells. In addition, photodepletion experiments with the fluorescent protein EGFP reveal the presence of a photoconversion process, which leads to a marked decrease in the brightness of the EGFP protein. We also identify conditions where the effect of EGFP's photoconversion on brightness experiments can be safely ignored.

## Introduction

Fluorescence correlation spectroscopy (FCS) and related techniques are well suited for the characterization of protein behavior in living cells [1], [2]. These fluorescence fluctuation spectroscopy (FFS) techniques rely on signal fluctuations of fluorescently labeled proteins passing through a small optical volume within the cell to characterize the sample. Auto- and cross-correlation methods are widely used to infer the mobility and interaction of the labeled proteins [2]–[5]. Another powerful application of FFS lies in the characterization of protein-protein interactions in living cells by brightness analysis [6]–[10] of homo-protein and hetero-protein complexes [8], [11], [12]. Consider a monomeric protein labeled with a fluorophore that creates a burst of photons as it passes through the observation volume. The average photon count rate of these bursts determines the molecular brightness of the labeled protein. Two labeled monomers that associate into a dimer result in a brightness twice that of the monomer, because the protein complex carries two fluorophores which produce, on average, twice the signal. This example illustrates that brightness encodes the average stoichiometry of protein complexes.

Analysis of FFS data requires caution as signal fluctuation can be affected in subtle but significant ways by the sample environment [13]. We report here that measurements of EGFP in yeast cells resulted in surprisingly large brightness scatter that was absent in mammalian cells measured under otherwise identical conditions. The cause of the scatter in brightness values is the cumulative, but subtle reduction of the fluorophore concentration by photobleaching, which we will refer to as photodepletion henceforth.

Less than 10% of photodepletion can introduce a bias in brightness of over 100%. This bias is problematic, because it obscures the correct interpretation of protein interaction data. We found that the impact of photodepletion on brightness depends strongly on the concentration of the fluorescently labeled protein. A simple model explains the brightness bias and identifies experimental conditions where photodepletion is of concern. We further describe segmented brightness analysis to effectively eliminate the influence of photodepletion on brightness data and verify it experimentally using a monomeric and dimeric fluorescent protein construct.

Closer inspection of the data over a wide range of photodepletion fractions for EGFP revealed the presence of photoconversion in addition to photobleaching. The photoconversion leads to a significantly reduced brightness of the EGFP protein. Although the presence of photoconversion complicates quantitative fluorescence experiments, we demonstrate that under most conditions its effect on brightness experiments is negligible. Thus, segmented brightness analysis offers a robust method to investigate protein interactions in the presence of photodepletion.

Our results reveal that brightness experiments in small sample compartments are vulnerable to photodepletion artifacts. The decay in the fluorescence intensity violates the implicit assumption of a stationary signal that forms the basis of conventional FFS theory. We broaden FFS theory by rigorously including the non-stationary photodepletion process. This enhanced formulation of FFS provides a framework for brightness experiments not only in yeast cells, but also in other small compartments, such as cellular organelles or bacterial cells, and extends the reach of brightness experiments significantly.

## Materials and Methods

### Experimental setup

The beam of a mode-locked Ti-Sapphire Laser (Mai-Tai, Spectra Physics, Mountain View, CA) serves as source for two-photon excitation. The laser light passes through either a 63× C-Apochromat water immersion objective (NA = 1.2, Zeiss, Thornwood, NY) or a 63× Plan Apochromat oil immersion objective (NA  =  1.4, Zeiss), and then excites the sample mounted on a modified Axiovert 200 microscope (Zeiss). Fluorescence emission light collected by the objective is transmitted through optical filters (Chroma Technology, Rockingham, VT) and detected by an avalanche photodiode (SPCM-AQ-14, Perkin-Elmer, Waltham, MA) in photon count mode. Two-photon FFS measurements on GFP samples were conducted at either 1000 nm or 905 nm with an excitation power of ∼1 mW as measured at the objective. Alexa-488 solution was measured with the same power at a wavelength of 900 nm. Photon counts were recorded into computer memory by a data acquisition card (PP1000, Celoxica, UK) for further analysis. Z-scan intensity profiles were carried out with a PZ2000 piezo stage (ASI, Eugene, OR) by moving the sample in the z-direction, which is parallel to the beam path. This scanning motion is controlled by an Agilent 33522A arbitrary waveform generator (Agilent Technologies, Santa Clara, CA) running a linear ramp signal with a frequency of 200 mHz and a peak-to-peak amplitude of 1.0 V. This voltage corresponds to an axial travel distance of ∼10.0 µm. One-photon photobleaching was conducted with a FluoArc mercury lamp (Zeiss) run between 80% and 100% power with light filtered by a (450–490 nm) optical bandpass filter (Chroma Technology). The spectrum of EGFP was measured with an Acton SP-2150i spectrograph (Princeton Instruments, Acton, MA) connected to an iXon 897 camera (Andor Technology, Belfast, UK).

### Microdroplets

A volume of 100 µL of Alexa-488 (Molecular Probes, Eugene, OR) dissolved in water was combined with 900 µL of silicon oil (Fisher Scientific, Fairlawn, NJ), pipetted for 5 seconds and then vortexed for 20 seconds. The emulsion was allowed to stand for three minutes while the larger droplets settle before removing a few μL from the top and transferring it onto a glass slide. A coverslip was pressed down on top and affixed at the corners with nail polish. FFS data were collected in the presence of photodepletion after focusing the two-photon spot at the center of the droplet.

### Yeast expression vector, cell line, sample preparation, and experimental protocol

Yeast strain of the EGFP vector, derived from the base S288C, was grown in a synthetic medium containing 2% raffinose overnight at ∼23 C°. For the expression of EGFP, galactose was added to the yeast culture (∼2% final concentration) when the optical density (OD) was about 0.4∼0.5 at 600 nm. When the OD reached 0.7∼0.8, the yeast culture was spun down (3000 G, 30 s) and resuspended with fresh synthetic medium. After repeating this step twice the concentration of yeast cells was concentrated 5X through resuspension in a reduced volume of medium. The concentrated yeast medium was mixed with low-temperature agar (1% final concentration) at ∼30 C°, and 2 µl of the mixture was pipetted on a microscope slide containing 5-μm microspheres that act as a spacer. The microscope slide was covered with a cover slip, and the slide's borders were sealed with nail polish. Sample preparation and culturing of yeast strain 3165 (described in [1]) expressing the dimeric construct EGFP_2_ was identical to the procedure above except that cells were grown in synthetic medium containing 2% glucose. Yeast cells were identified in bright field microscopy. We carefully selected a measurement position that avoided the nucleus and vacuoles, and took a z-scan measurement. Following that, FFS data were collected with a stationary beam focused into the cell.

### Mammalian expression vectors, cell Lines, sample preparation, and experimental protocol

U2OS, COS-1, MRC-5 and CV-1 cells (American Type Culture Collection, Manassas, VA) were transfected with either an EGFP-C1 plasmid or a tandem dimeric EGFP (EGFP_2_) plasmid as described previously [8]. These mammalian cells were maintained in a mixture of DMEM medium and 10% fetal bovine serum (Hyclone Laboratories, Logan, UT). U2OS, CV-1, COS-1 and MRC-5 cells were transfected using TransFectin reagent (Bio-Rad, Hercules, CA) according to the manufacturer's instructions 24 hours before measurement. All cells were subcultured into eight-well coverglass chamber slides (Nalge Nunc International, Rochester, NY) with the media replaced by Leibovitz L15 medium (Gibco, Auckland, NZ) immediately before measurement. FFS measurements on cells were performed as previously described [14]. For photodepletion experiments, cells were exposed repeatedly for short time intervals to epifluorescence light. After each exposure the instrument performed a short two-photon FFS measurement to record the brightness and the photodepletion fraction.

### Data analysis

Photon count data collected at a frequency of 20 kHz were analyzed with code written in IDL 8.0 (Research Systems, Boulder, CO). The brightness 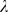 of the sample was determined by photon counting histogram (PCH) analysis and photon count moment analysis, in which deadtime and afterpulsing effects were corrected as previously described [6], [15]–[18]. The brightness was further corrected for the finite thickness of the cell by analysis of the z-scan intensity profile [13]. We measured the brightness 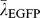 of EGFP either in water or in the nucleus of U2OS cells to establish a reference brightness for the fluorescent label. The standard deviation (SD) of the reference brightness was less than 10%. The normalized brightness of a yeast cell measurement is 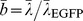. A dimeric protein carrying two EGFPs is represented by a normalized brightness 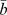 = 2, while a monomeric protein results in 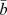 = 1. We use a bar over the symbol to stress that brightness is calculated from a time-average and not an ensemble average. The photodepletion rate coefficient 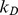 was determined from a fit of the intensity trace 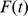 to a decaying exponential function, 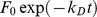, where 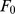 is the initial intensity. The photodepletion fraction 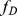 was calculated from the fluorescence intensity trace by 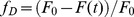. In segmented data analysis the photon count data was sliced into segments with a time interval *T.* Brightness 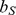 was calculated independently for each segment. We noticed the presence of undulations in some intensity traces from yeast cells. Such data was discarded, because it likely reflects the motion of vacuoles into and out of the excitation volume or the presence of focus drift during the measurement.

## Results

**Figure 1:**
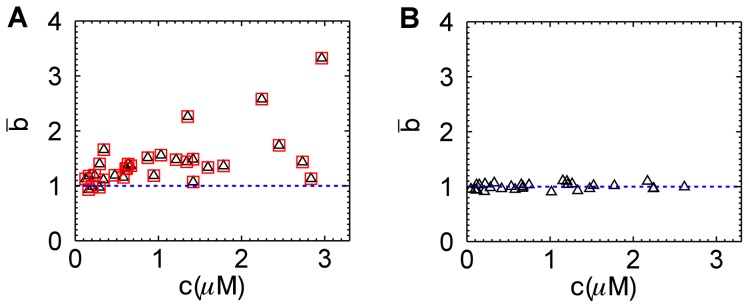
Normalized brightness of EGFP versus concentration. (A) EGFP in yeast cells results in brightness values that scatter from 1 to 3. Brightness is analyzed by PCH analysis (triangles) and by moment analysis (squares). (B) EGFP in U2OS cells exhibits a brightness close to 1 at all concentrations as expected for a monomeric protein. The blue dashed line represents the brightness value expected for monomeric EGFP.

We performed FFS experiments on yeast cells expressing EGFP by focusing the laser beam into the cytoplasm and collecting the fluorescence signal. After completing the FFS measurement an intensity z-scan was carried out to identify the thickness of the cytoplasmic layer at the measurement position as previously described [13]. The brightness 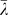 of the sample was determined by PCH analysis corrected for the axial thickness at the measured location [6], [15]. We converted it to the normalized brightness 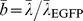 with the help of the reference brightness 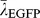. For convenience, we hereafter refer to normalized brightness simply as brightness. The analysis also identified the number of EGFP molecules in the optical observation volume, which was converted into a molar concentration. Since the amount of expressed EGFP differed between cells, repeating the experiment on many different yeast cells established the brightness over a wide concentration range. The result of this experiment, shown in Figure 1A, revealed an unexpected finding. While the brightness 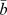
equaled one at low concentrations, as expected for a monomeric EGFP protein, the brightness at higher concentrations scattered between one and three. Brightness values larger than one indicate association between EGFP proteins [8]. However, EGFP is known to be monomeric at or below micromolar concentrations [8], [13], [19], as illustrated in Figure 1B, which depicts monomeric brightness values for EGFP measured in mammalian cells under the same experimental conditions as the yeast experiment. The brightness data shown in Figure 1A and B were determined by PCH analysis. As an additional check we reevaluated these data with an alternative analysis method based on photon count moments [16]–[18], which returned brightness values that are within a few percent identical to the PCH generated values (Figure 1A).

**Figure 2:**
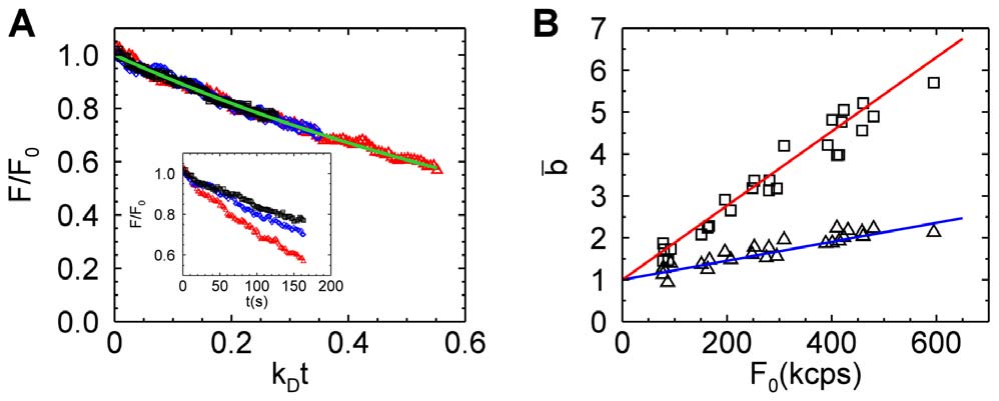
Fluorescent intensity decays in yeast cells and its effect on brightness values. (A) The fluorescent intensity decay from three different cell experiments (black, blue, and red symbols) is graphed versus the scaled time 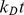. An exponential decay function 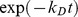 (green line) describes the experimental fluorescent intensity curves. The decay rate coefficient 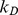 of the three cells differ (inset). (B) Brightness versus the initial intensity for data with a photodepletion fraction 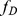 = 0.1 (triangles) and 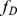 = 0.2 (squares). Modeling by Eq. 4 with photodepletion fractions of 0.2 and 0.1 is shown as the red and blue solid line, respectively.

We suspected that an experimental artifact was responsible for the difference in results between yeast and U2OS cells. Close inspection of the data revealed a small decrease (≤10%) in the intensity over the 30-second measurement period, which did not occur in the mammalian cell measurements. Because we anticipated that photobleaching plays a role, we performed another set of yeast experiments with much longer data acquisition times. The new data show a pronounced intensity decay with time (inset of Figure 2A). Fitting an exponential decay of the form 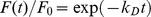 to the intensity traces determined the depletion rate coefficient 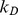, where 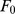 is the fluorescence intensity at the start of the experiment. The photodepletion rate of yeast cells varied (inset of Figure 2A). Larger yeast cells had a lower depletion rate than small cells, because it takes longer to deplete a large reservoir than a small one. Normalizing the fluorescence intensity trace to an amplitude of one and a rescaled time with respect to the depletion rate coefficient 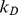
_,_ mapped all intensity traces to the same functional shape (Figure 2A). The relative decrease in fluorescence intensity is characterized by the photodepletion fraction 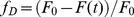. We calculated brightness 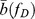 from fluorescence data as a function of the photodepletion fraction 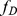 by truncating the fluorescence data at the point where the relative fluorescence decrease equaled the desired photodepletion fraction 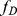. The brightness 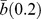 calculated from yeast data truncated at a photodepletion fraction of 0.2 depended strongly on the initial fluorescence intensity 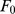 (Figure 2B, squares). If we instead calculated the brightness 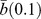 from a shorter segment of the data, so that the photodepletion fraction is only 0.1, we still observed a strong dependence of brightness on intensity (Figure 2B, triangles), but it was less pronounced than for the case 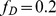.

The above observation demonstrated a link between the observed brightness bias and photodepletion. Larger photodepletion led to a stronger bias as seen in Figure 2B. However, even if the photodepletion fraction was kept the same, the bias was not constant, but depended on intensity. At low intensities the bias was almost negligible, while substantial at high intensities even for depletion fractions as low as 0.1. Thus, identifying the presence of photodepletion bias in brightness experiments seems important in order to avoid misinterpretation of data. Conventional FFS theory cannot predict the magnitude of this artifact, because it assumes a stationary fluorescence signal, which is violated in the presence of photodepletion. To account for the non-stationary signal in brightness calculations we consider a single photobleaching step converting the fluorescent protein from a fluorescent state *F* to a non-fluorescent dark state *D* with a rate coefficient that depends on the fluorophore and excitation light. Such a process leads to an exponential decay of the fluorescence intensity with time. At each time point 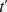
along the intensity trace a well-defined ensemble-averaged moment of the fluorescence intensity exists. The first and second ensemble-averaged moments are 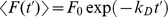 and 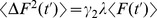, which utilizes the relationship between brightness and the first two intensity moments, 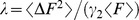 [6], [20]. The FFS experiment determines time-averaged moments with the first time-averaged moment 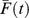 given by 
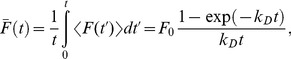
(1)where *t* represents the measurement time. All properties based on time-averaged moments will be denoted by a bar over the symbol. Applying the same procedure to the second central moment leads to 
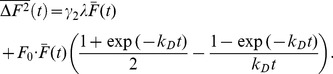
(2)


A detailed derivation of Eqs. 2 to 4 is found in Text S1. It is convenient to rewrite the above equations in terms of the photodepletion fraction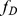, 
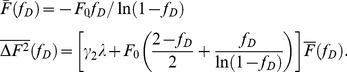
(3)


The time-averaged brightness 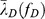 in the presence of photodepletion is determined by 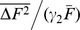 as 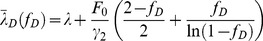
(4)


The above equation demonstrates that the time-averaged brightness 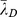 is larger than the ensemble-averaged brightness 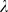, if photodepletion is present.

**Figure 3:**
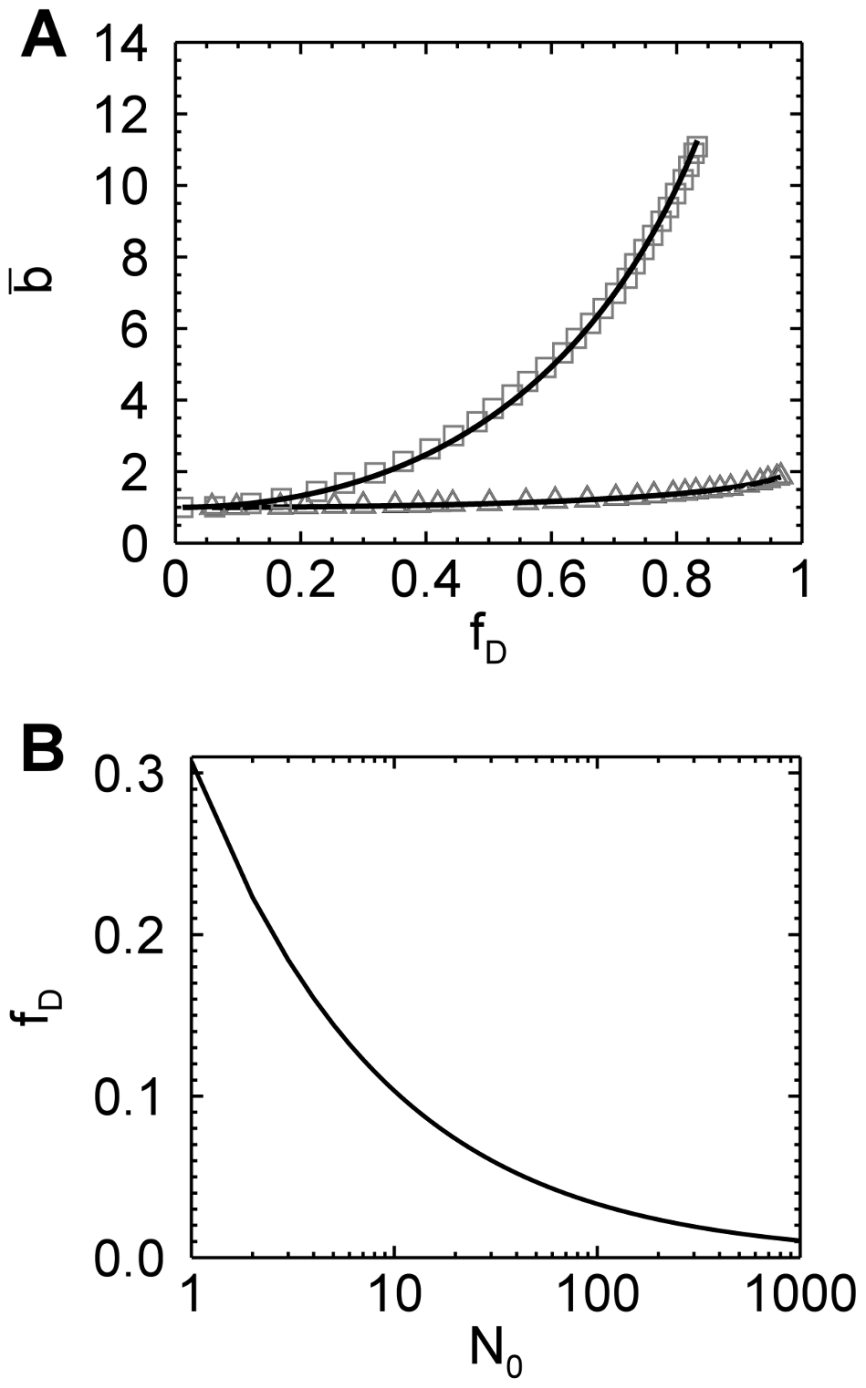
Time-averaged brightness bias. (A) The time-averaged brightness of Alexa488 as a function of the photodepletion fraction 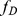 as measured in a microdroplet at a high (squares) and a low (triangles) concentration. The increase in brightness with 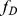
is an artifact caused by photodepletion. The solid lines represent the fit of the data to Eq. 4, which resulted in *N*_0_ of 16 and 0.69 for the high and low concentration data, respectively. (B) The relationship between photodepletion fraction 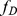 and the initial number of fluorescent molecules *N*_0_ in the optical observation volume that result in a brightness error of 5%.

Eq. 4 was tested using microdroplets containing Alexa488 solution embedded in silicon oil. Individual droplets were measured for a long enough time period to achieve photodepletion fractions in excess of 80%. Data were analyzed by systematically truncating the data at different lengths to vary the photodepletion fraction 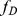 continuously. The brightness 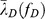 was divided by the reference brightness 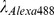 obtained from a measurement of a dye solution to get the normalized brightness 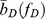. Figure 3A shows the brightness 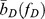 from two droplets, one containing a high concentration of dye and the other containing a very low concentration of dye, as a function of 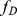, together with their respective fits to Eq. 4. The agreement between data and fits validated the simple model in an aqueous solution environment even for brightness biases as large as several hundred percent. We also tested the photodepletion model on cellular data by reexamining the brightness data from yeast cells shown in Figure 2B. Dividing Eq. 4 by the reference brightness 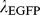 determined the time-averaged normalized brightness 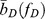. We plot 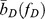 using photodepletion fractions of 0.2 and 0.1 as solid lines in Figure 2B and achieved excellent agreement with the experimental data.

After establishing the validity of the simple photodepletion model we investigated the influence of concentration on the brightness bias. The data in Figure 3A demonstrate that the sample with *N*_0_ = 16 dye molecules in the observation volume was very susceptible to photodepletion, while at the single molecule level (*N*_0_ = 0.69), the bias was only noticeable at very high photodepletion fractions. *N*_0_ describes the initial number of fluorescence molecules in the optical observation volume before photodepletion occurred. This dependence on concentration is also predicted by Eq. 4, because the initial intensity is proportional to the number of molecules, 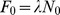. The relative error or bias can be written as,
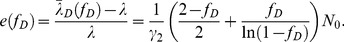
(5)


This formula is very convenient for estimating the potential bias of a brightness experiment. The initial number of molecules and the photodepletion fraction are the only factors needed to estimate the bias. Since the experimental uncertainty of brightness experiments is ∼10%, it is reasonable to set a bias limit that is half of the experimental uncertainty to ensure the absence of noticeable artifacts in brightness data. Eq. 5 was solved numerically for *e* = 5% to determine the limiting photodepletion fraction 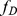 as a function of the initial number concentrations *N*_0_ (Figure 3B). The photodepletion fraction that guarantees a bias of 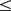
5% decreases with increasing concentration *N*_0_. Because fluorescence fluctuation experiments at concentrations higher that 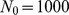 are rarely feasible, a photodepletion fraction of 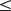
1% guarantees that brightness experiments in cells are free of the photodepletion artifact (Figure 3B). For reference, the highest concentration measured in this study is 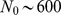. Since the fastest photodepletion rate coefficient obtained from the yeast cells is ∼0.006 s^−1^, a data segment length of ∼1.6 s guarantees a photodepletion fraction of 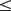
1%.

**Figure 4:**
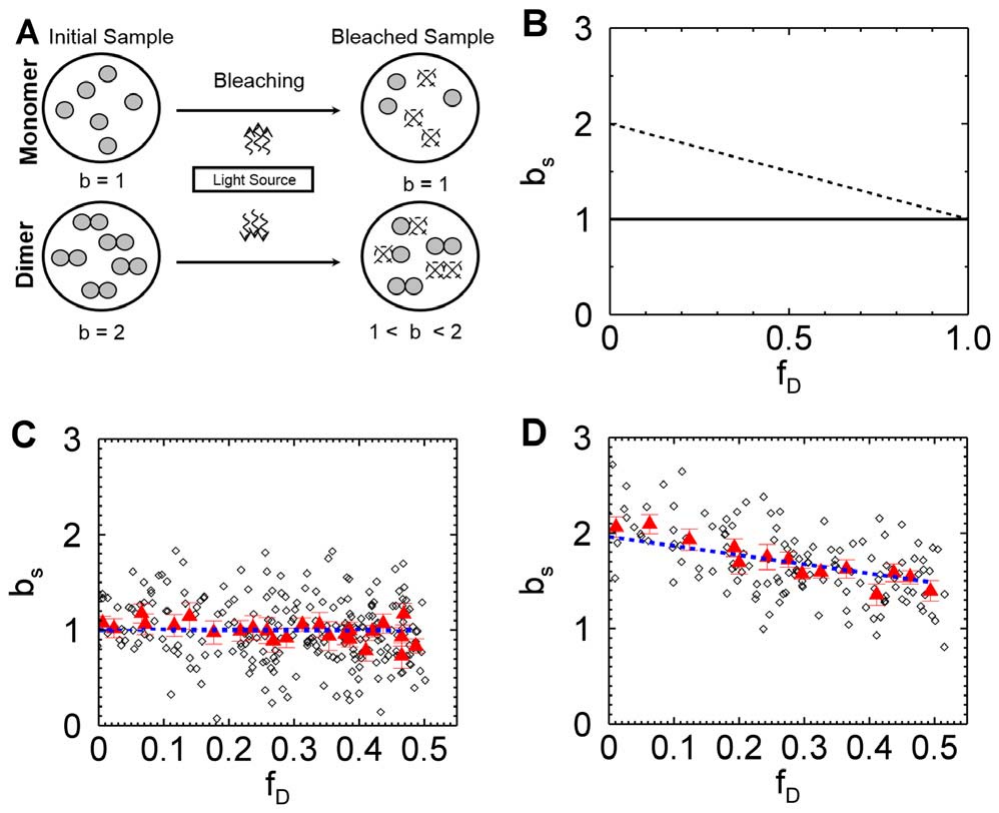
Segmented brightness analysis of monomers and dimers. (A) Illustration of photodepletion for monomers and dimers. Fluorescent molecules are depicted as filled circles and photobleached molecules are pictured as broken circles. The normalized brightness of monomers (*b* = 1) remains unchanged by photodepletion, In contrast, photobleaching of a dimeric sample with initial brightness of 2 leads to a reduction of brightness as explained in the text. (B) Theoretical brightness based on segmentation analysis of a monomeric (solid line) and dimeric (dashed line) sample as a function of photodepletion. (C) Brightness of EGFP from a yeast cell by segmentation analysis versus photodepletion fraction. Brightness values (diamonds) for a data segment size of 1.6 s. Ten consecutive brightness values are averaged (red triangles) to better visualize the trend of the data. The dashed blue line represents a fit of the brightness values to Eq. 6 with a fitted value of 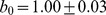. (D) Brightness of EGFP_2_ from a yeast cell by segmentation analysis versus photodepletion fraction. Symbols are described under (C). The blue dashed line represents a fit of the brightness values to Eq. 6 with a fitted value of 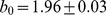.

Thus, it seems that dividing the data into sufficiently short segments provides a simple remedy to avoid artifacts due to photodepletion. However, photodepletion not only affects brightness through the introduction of a non-stationary signal, but also alters the brightness of oligomeric protein complexes. This is readily demonstrated by taking a closer look at the photobleaching process (Figure 4A). We assumed a simple model wherein a fluorescent protein *F* with normalized brightness *b* = 1 is irreversibly converted into a non-fluorescent state *D* with brightness zero. Consider first the case of a monomeric protein *F*. Photobleaching leads to two populations, *F* and *D*. Only state *F* contributes to the fluorescence signal. Because each protein in state F has the same brightness, photobleaching has no effect on the brightness of the sample. A population of dimers *F*_2,_ on the other hand, initially has a normalized brightness of 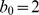. Photobleaching introduces three distinct species that differ in their brightness (Figure 4A). If both fluorophores of the dimer are photobleached (state *D*_2_), then the complex is dark with a brightness of zero. If both fluorphores survive (state *F*_2_), the brightness of the complex remains that of a dimer. If one of the two fluorophores survives (state *FD*), the complex has a brightness of 1. This mixture of brightness states leads to an apparent brightness between 1 and 2. Since the population of states *FD* and *D*_2_ increases with time, the brightness of the dimer decreases in the presence of photodepletion [8].

Let us explicitly treat the case of a population of *n*-mers *F*_n_. Bleaching of *s* chromophores leads to the species *F*_n-s_ *D*_s_ with normalized brightness 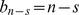. Since photobleaching of individual chromophores is statistically independent, the probability *p* of a single chromophore to be bleached equals the photodepletion fraction, 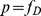. Thus, the probability of an n-mer to be in state *F*_n-s_ *D*_s_ is given by 
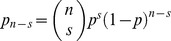
. The sample consists of a mixture of populations *F*_n-s_ *D*_s_, which leads to an average or apparent normalized brightness of [8]

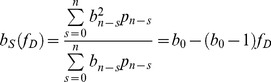
(6)where 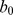 represents the initial normalized brightness of the sample in the absence of photodepletion. Note that Eq. 6 specifies the brightness 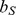 from a short data segment of a sample with a photodepletion fraction 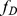. For an *n*-mer the initial normalized brightness is 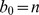. The behavior of Eq. 6 is illustrated in Figure 4B for a dimer (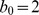) and monomer (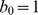) sample. The normalized brightness starts at a value of 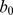 in the absence of photodepletion (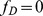) and decreases linearly to a value of one as the photodepletion fraction approaches one. This result reflects that the last surviving fluorescent population of an *n*-mer is *F*_1_ *D*_n-1_ with exactly one remaining fluorophore, which implies a normalized brightness of one. Of course, Eq. 6 also predicts that the brightness of a monomeric protein sample stays constant as discussed earlier. While we derived Eq. 6 for a homogenous sample of *n*-mers, it is straightforward to show that the equation remains correct for a mixture of oligomeric states with 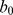 representing the apparent brightness of the mixture. Note that we removed the bar over the brightness symbol to emphasize that the segmented brightness equals the ensemble-averaged brightness.

We performed segmented brightness analysis on data taken from a yeast cell expressing EGFP. The brightness of each segment is graphed as a function of the photodepletion fraction (Figure 4C). The brightness values showed significant scatter reflecting the poor statistics due to the short segment size of 1.6 s. We also graph the brightness averaged over 10 segments, which reduces the scatter and aids in visualizing data trends. The initial brightness of the cell was determined by a fit of the segmented brightness values with Eq. 6. The fit (dashed line, Figure 4C) resulted in a brightness of 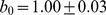 (reduced Chi-squared = 1.0), as expected for monomer EGFP. Next, we examined a dimeric fluorescent protein by expressing the tandem construct EGFP_2_ in yeast cells. The data was subjected to the same analysis as described above. The segmented brightness appeared to diminish with depletion fraction (Figure 4D), which is a trend predicted by the model (Figure 4A & B). The dashed line describes the fit of the data to Eq. 6 with an initial brightness 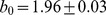 (reduced Chi-squared = 1.1), which is consistent with dimeric EGFP.

While fitting of the segmented brightness values by Eq. 6 is feasible, there is a simpler alternative. The average of all segmented brightness values of the experiment, 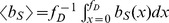 is, according to Eq. 6, related to the initial brightness, 
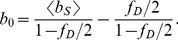
(7)


We used Eq. 7 to compute the initial brightness 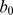 for the data shown in Figures 4C & D, which yielded 0.98 for EGFP and 1.93 for EGFP_2_. These values agree with the results from the earlier analysis based on Eq. 6. However, Eq. 7 is more convenient, because no fitting is required.

**Figure 5:**
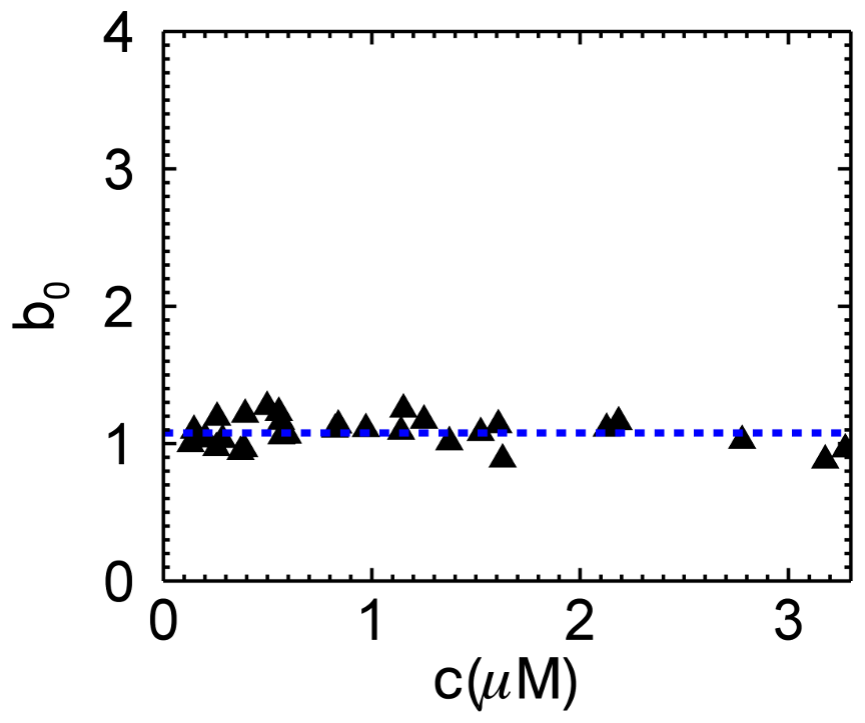
Normalized brightness of EGFP versus concentration in yeast cells. The same data shown in Figure 1A are reanalyzed with segmented brightness analysis, which removes the bias (mean and SD of brightness data: .1.08±0.10).

We performed segmented brightness analysis on the yeast data previously shown in Figure 1A using a segment length of 1.6 s. The initial brightness 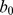 was calculated with Eq. 7 to determine the initial brightness (Figure 5). We see that the new analysis successfully removed the earlier brightness scatter (Figures 1A) and produced a brightness 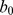 close to one (mean and SD: 1.08±0.10), which were in good agreement with the result obtained for mammalian cells in Figure 1B (mean and SD: 1.01±0.06).

**Figure 6:**
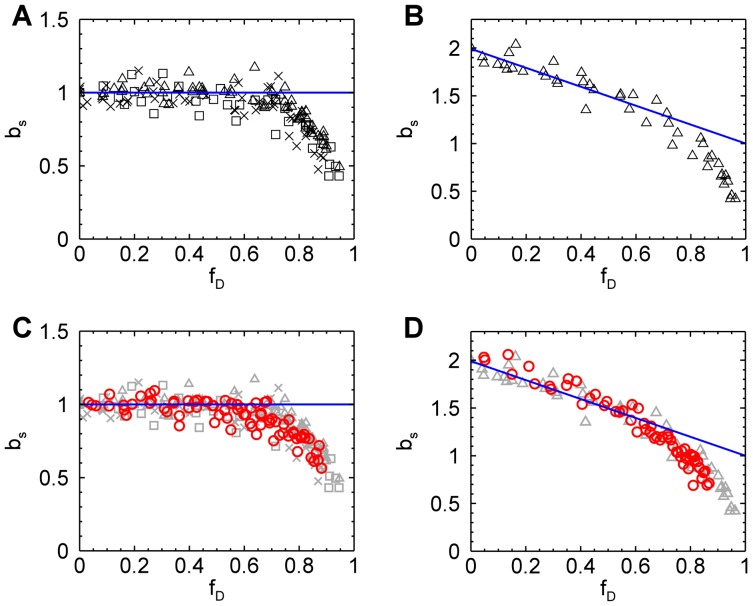
Segmented brightness of EGFP and EGFP_2_ in mammalian and yeast cells versus photodepletion fraction. Photobleaching of mammalian cells was accomplished by one-photon excitation, while yeast cells were photobleached by two-photon excitation. (A) The normalized brightness of EGFP in mammalian cells (five COS cells (triangles), five CV-1 cells (squares), six MRC5 cells (crosses)) is close to the theoretical value (blue line) until the photodepletion fraction exceeds 0.6, at which point it begins to drop. (B) The normalized brightness of EGFP_2_ in mammalian cells agrees with theory (blue line) until the photodepletion fraction reaches 0.6. (C) The normalized brightness of EGFP from three yeast cells (red circles) shows the same behavior as seen in mammalian cells (grayed symbols, same as shown in panel A). (D) The normalized brightness of EGFP_2_ from two yeast cells (red circles) close follows the brightness observed in mammalian cells (grayed symbols, same as shown in panel B).

While the above results demonstrate that quantitative brightness analysis in the presence of photodepeletion is feasible, we have not yet examined the range of photodepletion fractions covered by our model. This is especially important for EGFP, since the photophysics of GFP-like proteins is remarkably complex [21], [22], while our model is based on a single photobleaching step. To address this question we performed extended photodepletion experiments both in mammalian and yeast cells as described in the Materials and Methods section to achieve photodepletion fractions in excess of 80%. The segmented brightness of several mammalian cells expressing EGFP is graphed as a function of the photodepletion fraction (Figure 6A). The segmented brightness initially remained at one, as expected for a monomer (Eq. 6). However, once the photobleaching fractions exceeded 60% a decrease in the segmented brightness is noted. This result indicates that our bleaching model is too simplistic. Analogous photodepletion experiments were also performed on mammalian cells expressing the tandem construct EGFP_2_. The segmented brightness values closely followed the curve (blue line) expected for a dimer for 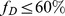 (Figure 6B), but was falling off faster than predicted by theory for photodepletion fractions exceeding 60%.

We further conducted extended photodepletion experiments on yeast cells to identify whether the unexpected brightness behavior at high photodepletion fractions of mammalian cells was also found in yeast cells. Figure 6C shows the averaged segmented brightness (red circles) from several yeast cells expressing EGFP together with the earlier results obtained from mammalian cells. The corresponding data (red circles) from yeast cells expressing EGFP_2_ are graphed together with the results from mammalian cells in Figure 6D. We observed an identical response of segmented brightness with photodepletion fraction for yeast and mammalian cells.

Our model is based on a photobleaching reaction from a single bright to a non-fluorescent state, 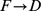. The data show that the brightness behavior of EGFP was well approximated by this simple model provided the photodepletion fraction was less than 60%. However, the decrease in brightness of monomeric EGFP for 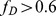, necessitates the appearance of a second brightness state, which we denote as 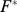. This new state is likely populated by a photoconversion process, as explained later, and has to be less bright than the original state *F* to explain the drop in brightness observed in the data.

Photoconversion of EGFP from a green to a red fluorescent state by an electron transfer process has been recently reported [23], [24]. We measured the fluorescence emission spectrum of a U2OS cell before and after photobleaching to identify whether the appearance of state 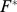 is associated with a strong shift in the emission spectrum towards the red. The emission spectrum after strong photodepletion (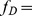
0.87) was virtually identical to the emission spectrum of the unbleached sample. Because state *F* and 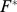
are both green fluorescent states, the observed photoconversion process is distinct from the reddening of EGFP. We also measured the fluorescence lifetime in the absence (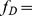
0) and presence (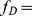
0.77) of photodepletion in U2OS cells (see Text S3). The time-resolved fluorescence intensity decay is close to a mono-exponential for 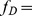
0, while photodepletion leads to the appearance of a second, shorter lifetime component, which is responsible for the initial faster decay of the intensity trace (Figure S1). The change in the time-resolved fluorescence decay with photodepletion supports the existence of a photoconverted EGFP state as suggested by the brightness experiments.

## Discussion

Because correcting photobleaching effects is not straightforward, most FFS experiments use conditions where the probability of a fluorophores being photobleached as it passes through the laser beam is sufficiently small to not directly distort the statistics of the fluctuations. The occasional photobleaching event still reduces the number of fluorophores in the sample. This reduction has a negligible effect on concentration for sufficiently large sample reservoirs. However, this is not the case for small sample volumes, where the cumulative effect of photobleaching leads to a measurable reduction in the concentration of fluorophores over the measurement period. The budding yeast *Saccharomyces cerevisiae* is spherical-shaped with a diameter of ∼5 µm. Its volume of ∼60 fL is significantly smaller than that of a mammalian cell with a volume of a few pL. The data in Figure 1 demonstrate that photodepletion which is negligible for mammalian cells, cannot be ignored for budding yeast cells under identical experimental conditions. Photodepletion effects are also variable within a given cell population. While the median cell size of budding yeast depends on species and strain, there is significant variability in cell size in any given population of yeast, which gives rise to the differences in the observed photodepletion rates (inset, Figure 2A).

The analysis of FCS and related techniques is based on a stationary fluorescence signal, which is violated in the presence of photodepletion. We developed a model that explicitly takes the non-stationary signal due to the depletion of fluorophores into account. This model explains the observed brightness bias of conventional analysis (Figures 2B and 3A). It further predicts the linear relation between bias and initial sample concentration (Eq. 5), which explains the absence of significant brightness error at low concentration. For example, a photodepletion fraction of 10% leads to a bias of less than 20% for concentration < 200 nM with a focal volume of 0.2 fL. These conditions were met in an earlier study reporting the first brightness measurements in *S. cerevisiae* [1]. However, the analysis bias is not negligible at higher concentrations, and artificially increased brightness values would lead to an erroneous conclusion about protein complex formation.

What factors are responsible for the observed increase in brightness when photodepletion occurs? Conventional theory states that for a stationary process the brightness is proportional to the ratio of variance to mean of the fluorescence, 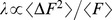 [6], [20] Photodepletion increases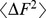, because the resulting intensity decrease constitutes an extra variation of the signal that is added to the intensity variations caused by fluorescent proteins diffusing in and out of the observation volume. By the same token, photodepletion decreases the mean fluorescence 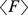. Both factors increase the ratio 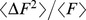, which explains the observed inflation of the brightness value.

Segmentation of the photon count data provided an effective strategy to eliminate biases due to photodepletion. The appropriate segment size is calculated using the photodepletion rate and Eq. 5. Because the segment size is short, the scatter in brightness is large (Figures 4C and D), and further data processing is necessary to identify the initial brightness of the sample. The segmented brightness 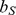 decreases linearly as a function of the photodepletion fraction 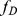 with a slope that depends on the initial brightness (Eq. 6). While fitting of the slope provides the unbiased brightness, we prefer to directly compute the initial brightness from Eq. 7. Applying this method to the measurements of EGFP in *S. cerevisiae* eliminates the scatter seen in Figure 1A and yields the expected brightness for monomeric EGFP at all concentrations (Figure 5). The standard deviation of segmented brightness analysis is ∼10% and represents a significant improvement over the uncertainty of previously reported brightness data in yeast [1], [25]. This value is close to the standard deviation achieved in mammalian cells.

The method introduced in this paper determines the maximum segment length that guarantees a relative brightness bias of 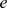 or less (for a brief summary of the protocol see Text S2). There also is a minimum length requirement, because enough fluctuations need to be sampled during a single segment to ensure a meaningful calculation of brightness. Based on our experience 100 independent fluctuations are sufficient to provide enough sampling for determining brightness. The diffusion time is a measure of the duration of a fluctuation. Since the number concentration *N* of experiments in cells is larger than one, a segment time of 100 diffusion times ensures the sampling of 100 independent fluctuations. Because the diffusion time of soluble proteins in cells is typically a few milliseconds, we estimate a minimum segment time of a few hundred milliseconds. Note that our analysis method is not applicable if the minimum segment time exceeds the maximum segment time. Such a situation may arise for slowly diffusing proteins, such as membrane proteins, and needs to be checked before applying segmented brightness analysis. In our case there is no concern, because a diffusion time of ∼1 ms for EGFP in yeast leads to a minimum segment length (∼100 ms), which is much shorter than the maximum segment length of 1.6 s.

**Figure 7:**
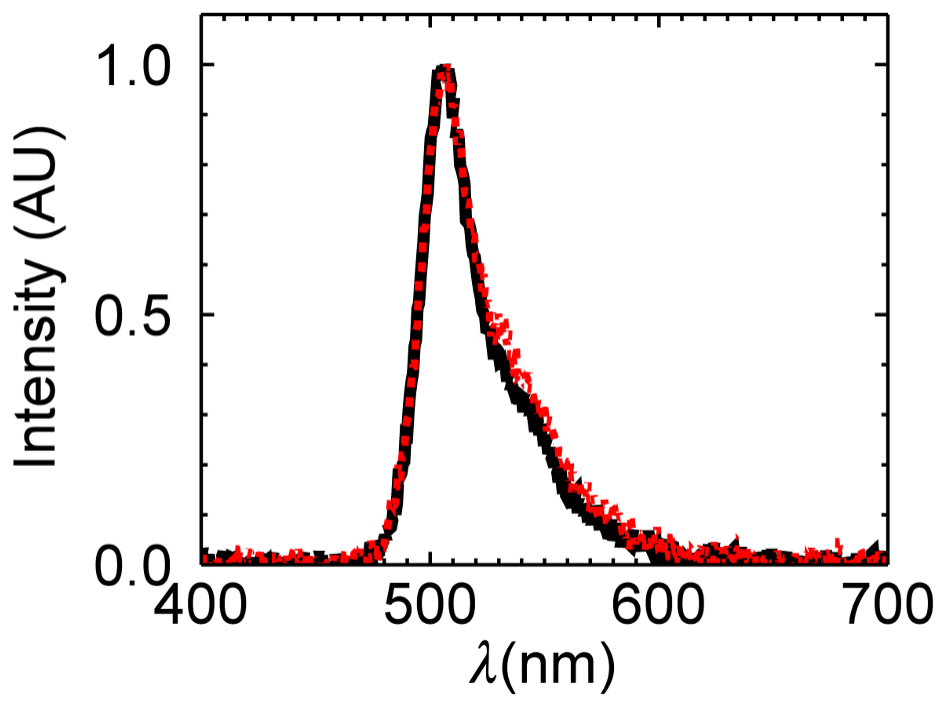
Fluorescence emission spectrum of EGFP before and after photodepletion. The initial spectrum (black line) before photodepletion is virtually identical to the spectrum (red line) taken at a photodepletion fraction of 0.87. Both spectra have been scaled to a maximum amplitude of one to facilitate visual comparison.

We assumed a simple photobleaching process that converts a fluorescent state *F* with normalized brightness 1 to a non-fluorescent state *D*. EGFP deviates from this simple model, because we observed a drop in the segmented brightness for 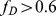 (Figure 5A). This behavior provides conclusive evidence that the state *F* is not the only brightness state of EGFP. For simplicity we consider just one additional state F^*^. As mentioned earlier this state must have a lower brightness than state *F*. It has been shown that the presence of a mixture of brightness states within a fluorescent protein leads to a dimeric brightness that is less than double [26], [27]. Thus, EGFP is initially well described by a single brightness state *F*, because we observed (Figure 6D) within experimental uncertainty brightness doubling for the dimeric EGFP construct [8], [28]. Because the lower brightness state F^*^ contributes less to the overall brightness of the sample compared to state *F*, the drop in brightness is not observed until a significant population of the fluorescent proteins is in state F^*^. Thus, the most likely explanation for the presence of a large population of F^*^ at large photodepletion fractions is the presence of a photoconversion process that populates state F^*^ in addition to the photobleaching process. This photoconversion process, however, is not associated with a change in the emission spectrum (Figure 7).

Because the photophysics of EGFP is complex [21], [22], identifying the exact nature of the state F^*^ will require additional studies. However, the presence of more than one state of EGFP is supported by additional experiments. One- and two-photon photobleaching studies of EGFP have reported non-exponential decay characteristics [29], [30], which support the existence of more than one state. Conversely, it has been argued that the non-exponential photobleaching kinetics might be explained by Lévy statistics [31]. However, single molecule studies have reported that occasionally two photobleaching steps are observed for single EGFP molecules [21], [32]. This observation is consistent with the presence of a second brightness state of EGFP.

We would like to stress that despite the appearance of a second brightness state, our analysis with the simple bleaching model is successful as long as the photodepletion fraction is less than ∼60%. Since this condition is met for virtually all FFS experiments, the segmentation method described in this paper provides a robust analysis method. Segmentation has been originally suggested to lessen the influence of cytoplasmic intensity drifts on brightness measurements [14] The same approach has been used to correct distortions in the autocorrelation functions caused by photobleaching [33]. However, the importance of data segmentation in brightness analysis has gone largely unnoticed [34]. For example, it is common to apply PCH analysis to the entire data set [8]. This paper provides the first quantitative formulation of segmented brightness analysis and a framework for future investigation of non-stationary processes by brightness. A novel aspect of this technique is the identification of a potential photoconversion process of EGFP by relying on brightness instead of emission color. The existence of a photoconversion process is further corroborated by changes in the fluorescence lifetime of EGFP. Thus, segmented brightness analysis could prove useful for providing insights into the behavior of EGFP and other fluorescent proteins that are difficult to obtain by other methods. The properties of EGFP are of particular interest, because EGFP serves as the fluorescent tag of a vast number of cellular studies. We expect that characterization of brightness conversion processes should prove important for fluorescence-based cellular studies. For example, stepwise photobleaching experiments count the number of fluorescently-labeled subunits in a protein complex [32], [35]. Photoconversion of the fluorophore into a different brightness state compromises the count statistics of the experiment.

While EGFP is relatively photostable, some other fluorescent proteins are much more photolabile. For example, photodepletion of a red fluorescent protein has been observed in two-photon FFS measurements in mammalian cells [26]. In addition, while photobleaching by two-photon excitation is strictly confined to the focal volume, photobleaching by one-photon excitation occurs also outside the focal volume, which potentially accelerates the appearance of photodepletion and its artifacts. Thus, photodepletion effects are potentially relevant not only for yeast experiments, but also for measurements in larger volumes, such as in mammalian cells.

The ability of FFS to perform brightness titrations is a powerful tool, but only if brightness can be correctly related to protein stoichiometry and concentration. This paper introduces a general theory for incorporating a non-stationary process into the analysis of fluorescence fluctuations. This expanded formulation of FFS was essential for the correct identification of brightness and concentration in the presence of photodepletion as demonstrated for the budding yeast *S. cerevisiae*. The new analysis approach should also prove useful for brightness experiments in other small compartments, such as cellular organelles or bacterial cells. We expect that the modified FFS theory provides a useful framework for future investigation of protein interactions of non-stationary processes in living matter by brightness techniques.

## Supporting Information

Figure S1 **Time-resolved fluorescence decay curve of EGFP in the presence and absence of photodepletion.**
(DOCX)

Text S1 **Derivation of time-averaged variance of the fluorescence intensity.**
(DOCX)

Text S2 **Protocol for brightness analysis in small sample compartments.**
(DOCX)

Text S3 **Fluorescence lifetime measurement.**
(DOCX)
