# Supplementary material for: Quantitative Measurement of Brightness from Living Cells in the Presence of Photodepletion
Source: PLoS One. 2014 May 12;9(5):e97440. doi: 10.1371/journal.pone.0097440 (PMC4018325; doi:10.1371/journal.pone.0097440)
Supplement: Figure S1 — Time-resolved fluorescence decay curve of EGFP in the presence and absence of photodepletion. (DOCX) [file pone.0097440.s001.docx]

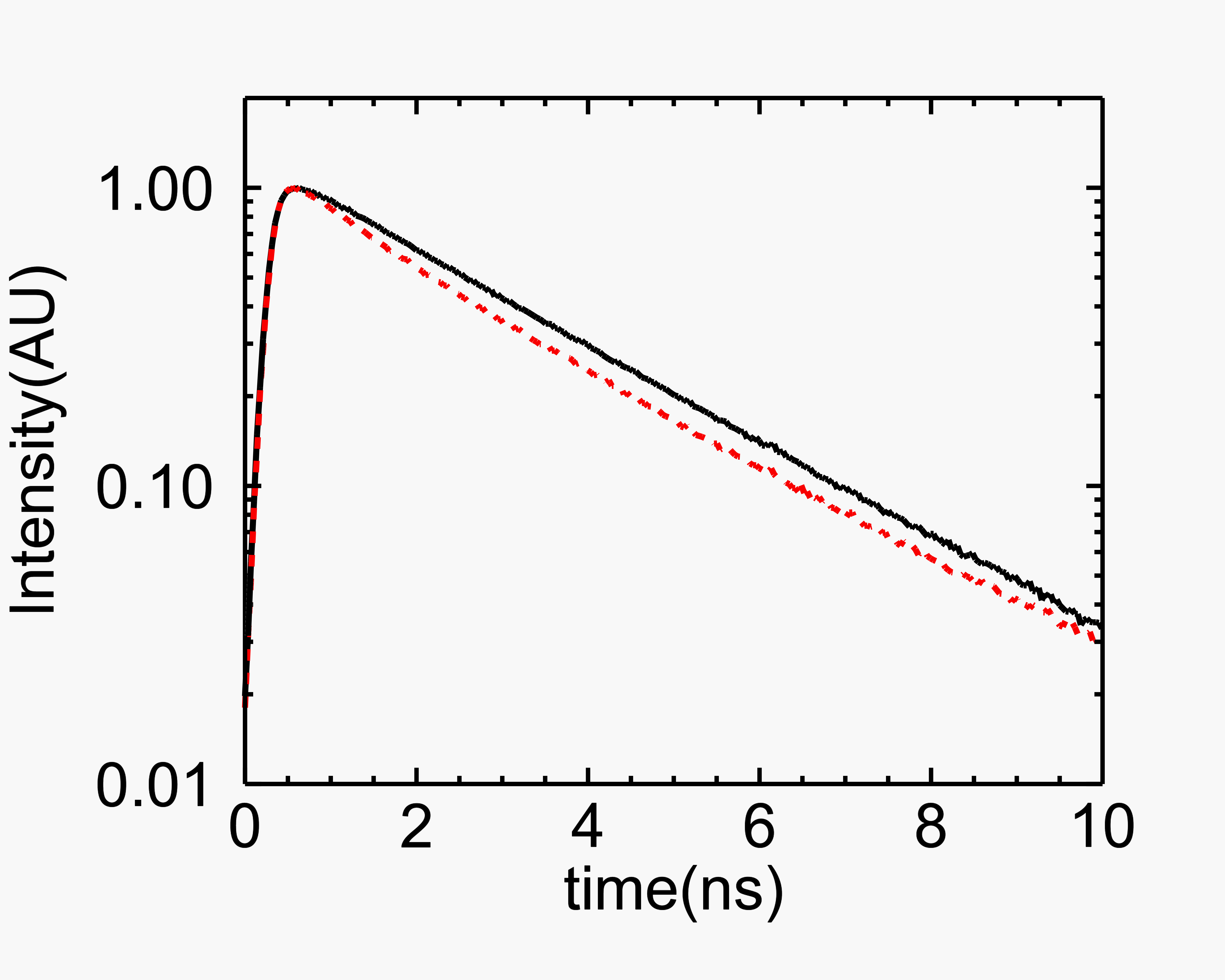


**Supporting Figure S1. Time-resolved fluorescence decay curve of EGFP in the presence and absence of photodepletion.** The time-resolved fluorescence decay curves of EGFP in the absence of photodepletion ($f_{D}=0$, black line) and in the presence of photodepletion ($f_{D}=0.77$, red line) are normalized to an amplitude of one to facilitate visual comparison. The decay in the absence of photodepletion is approximately described by a single exponential decay with a lifetime of ~2.6 ns. The fluorescence decay in the presence of photodepletion (red line) exhibits an initial fast decay, which reveals the presence of a short lifetime component. The slope of the decay at later times is roughly equal to the slope of the original decay curve (black line), indicting the presence of a second lifetime component of ~2.6 ns.
