# Supplementary material for: Quantitative Measurement of Brightness from Living Cells in the Presence of Photodepletion
Source: PLoS One. 2014 May 12;9(5):e97440. doi: 10.1371/journal.pone.0097440 (PMC4018325; doi:10.1371/journal.pone.0097440)
Supplement: Text S1 — Derivation of time-averaged variance of the fluorescence intensity. (DOCX) [file pone.0097440.s002.docx]

**Supporting Text S1 Derivation of time-averaged variance of the fluorescence intensity**

The time-averaged fluorescence $\overline{F}\left(t\right)$ for a measurement starting at time 0 and ending at time *t* is given by Eq. 1 of the main text. We define the fluctuation as the deviation of the instantaneous fluorescence $F\left(t'\right)$ at time $t'$ from the time-averaged fluorescence $\overline{F}\left(t\right)$, $\delta F\left(t'\right)=F\left(t'\right)-\overline{F}\left(t\right)$. The ensemble variance is determined from the fluctuations by $\left\langle\Delta F^{2}\right\rangle=\left\langle\delta F\left(t'\right)^{2}\right\rangle$, which for a non-stationary process is time-dependent. The FFS experiment determines the time-averaged variance by

$\overline{\Delta F^{2}}\left(t\right)=\frac{1}{t}\int_{0}^{t} \left\langle\delta F\left(t'\right)^{2}\right\rangle dt'=\frac{1}{t}\int_{0}^{t} \left\langle\left(F\left(t'\right)-\bar{F}\left(t\right)\right)^{2}\right\rangle dt'$. (S)

To evaluate the integral we first rewrite the integrand $\left\langle\left(F\left(t'\right)-\bar{F}\left(t\right)\right)^{2}\right\rangle$ in the form $\left\langle F^{2}\left(t'\right)\right\rangle-2\left\langle F\left(t'\right)\right\rangle\bar{F}\left(t\right)+\bar{F}\left(t\right)^{2}$ and apply the identity $\left\langle F\left(t'\right)^{2}\right\rangle=\left\langle\Delta F\left(t'\right)^{2}\right\rangle+\left\langle F\left(t'\right)\right\rangle^{2}$. Inserting the reformulated integrand into Eq. S yields

$\overline{\Delta F^{2}}\left(t\right)=\frac{1}{t}\int_{0}^{t} \left(\left\langle\Delta F^{2}\left(t'\right)\right\rangle+\left\langle F\left(t'\right)\right\rangle^{2}-2\left\langle F\left(t'\right)\right\rangle\bar{F}\left(t\right)+\bar{F}\left(t\right)^{2}\right)dt'$. (S)

By applying the sum rule and taking all constant factors out of the integral we get

$\overline{\Delta F^{2}}\left(t\right)=\frac{1}{t}\int_{0}^{t} \left\langle\Delta F^{2}\left(t'\right)\right\rangle dt'+\frac{1}{t}\int_{0}^{t} \left\langle F\left(t'\right)\right\rangle^{2}dt'-\bar{F}\left(t\right)\frac{1}{t}\int_{0}^{t} \left\langle F\left(t'\right)\right\rangle dt'$. (S)

Evaluating the integrals requires the ensemble-averaged intensity $\left\langle F(t')\right\rangle=F_{0}\exp\left(-k_{D}t'\right)$ and its variance $\left\langle\Delta F^{2}(t')\right\rangle=\gamma_{2}\lambda\left\langle F(t')\right\rangle$ as introduced in the manuscript. The three terms of Eq. S evaluate to

$\begin{matrix} \frac{1}{t}\int_{0}^{t} \left\langle\Delta F^{2}\left(t'\right)\right\rangle dt'=\frac{1}{t}\int_{0}^{t} \gamma_{2}\lambda\left\langle F\left(t'\right)\right\rangle dt'=\gamma_{2}\lambda\bar{F}\left(t\right) \\ \frac{1}{t}\int_{0}^{t} \left\langle F\left(t'\right)\right\rangle^{2}dt'=\bar{F}\left(t\right)F_{0}\frac{1+\exp\left(-k_{D}t\right)}{2} \\ \bar{F}\left(t\right)\frac{1}{t}\int_{0}^{t} \left\langle F\left(t'\right)\right\rangle dt'=\bar{F}\left(t\right)^{2} \end{matrix}$ (S)

The sum of the three terms simplifies to

$\overline{\Delta F^{2}}\left(t\right)=\gamma_{2}\lambda\bar{F}(t)+F_{0}\cdot\bar{F}(t)\left(\frac{1+\exp(-k_{D}t)}{2}-\frac{1-\exp(-k_{D}t)}{k_{D}t}\right)$, (S)

which is Eq. 2 of the manuscript.

It is advantageous to express $\bar{F}\left(t\right)$and $\overline{\Delta F^{2}}\left(t\right)$ as a function of the photodepletion fraction$f_{D}$ by using the relation $f_{D}=\left(F_{0}-F(t)\right)/F_{0}=1-\exp\left(-k_{D}t\right)$. By inserting $k_{D}t=-\ln\left(1-f_{D}\right)$ into Eqs. 1 and S, we recover Eq. 3 of the manuscript,

$\begin{matrix} \bar{F}\left(f_{D}\right)=-F_{0}f_{D}/\ln\left(1-f_{D}\right) \\ \overline{\Delta F^{2}}\left(f_{D}\right)=\left[\gamma_{2}\lambda+F_{0}\left(\frac{2-f_{D}}{2}+\frac{f_{D}}{\ln\left(1-f_{D}\right)}\right)\right]\bar{F}\left(f_{D}\right) \end{matrix}$. (S)

The time-averaged brightness $\bar{\lambda}_{D}\left(f_{D}\right)$ is determined from the above equation by

$\bar{\lambda}_{D}\left(f_{D}\right)=\frac{1}{\gamma_{2}}\frac{\overline{\Delta F^{2}}\left(f_{D}\right)}{\bar{F}\left(f_{D}\right)}=\lambda+\frac{F_{0}}{\gamma_{2}}\left(\frac{2-f_{D}}{2}+\frac{f_{D}}{\ln\left(1-f_{D}\right)}\right)$, (S)

which corresponds to Eq. 4 of the manuscript. As expected, the time-averaged brightness $\bar{\lambda}_{D}\left(f_{D}\right)$ reduces to the true brightness $\lambda$ in the absence of photodepletion ($f_{D}=0$) as easily verified by applying l'Hopital's rule to Eq. S.
