# Supplementary material for: Quantitative Measurement of Brightness from Living Cells in the Presence of Photodepletion
Source: PLoS One. 2014 May 12;9(5):e97440. doi: 10.1371/journal.pone.0097440 (PMC4018325; doi:10.1371/journal.pone.0097440)
Supplement: Text S2 — Protocol for brightness analysis in small sample compartments. (DOCX) [file pone.0097440.s003.docx]

**Supporting Text S2**

**Protocol for Brightness Analysis in Small Sample Compartments**

A prudent first step when analyzing data from a small sample compartment is testing whether photodepletion is present. The intensity trace of the experiment is fit to an exponential decay of the form $F(t)=F(0)\exp\left(-k_{D}t\right)$ to determine the depletion rate coefficient $k_{D}$ and the photodepletion fraction $f_{D}=\left(F(0)-F(T)\right)/F(0)$, where $F(0)$ and $F(T)$ represent the initial and final fluorescence intensity of the experiment. Next, choose the relative brightness bias $e$ that is experimentally acceptable. We suggest a value of $e=0.05$ as explained in the manuscript. The highest concentration that guarantees an error of $e$ or less is determined by Eq. 5 of the manuscript. Because concentration and initial fluorescence intensity are related, $F(0)=\lambda_{EGFP}N_{0}$, we rewrite Eq. 5 to determine the highest initial intensity $F_{\max}(0)$ that ensures a brightness error of $e$ or less,

$F_{\max}(0)=\gamma_{2}\frac{e}{\frac{2-f_{D}}{2}+\frac{f_{D}}{\ln\left(1-f_{D}\right)}}\bar{\lambda}_{EGFP}$, (S)

where $\bar{\lambda}_{\text{EGFP}}$ is the calibrated brightness of the fluorescent protein measured in a large sample reservoir. If the initial intensity of the experiments is less than $F_{\max}(0)$, then photodepletion can be safely neglected.

However, if the initial intensity $F(0)$ of the experiments exceeds $F_{\max}(0)$, the data need to be segmented to reduce the photodepletion fraction from $f_{D}$ to $f_{D}^{*}$. The value of $f_{D}^{*}$ is determined by graphically or numerically solving the equation

$F(0)=\gamma_{2}\frac{e}{\frac{2-f_{D}^{*}}{2}+\frac{f_{D}^{*}}{\ln\left(1-f_{D}^{*}\right)}}\bar{\lambda}_{EGFP}$. (S)

The segment length is determined by the time $T^{*}$ that results in a photodepletion fraction $f_{D}^{*}$,

$T^{*}=-\frac{\ln\left(1-f_{D}^{*}\right)}{k_{D}}$. (S)

Segmenting the data into time intervals of length $T^{*}$ followed by segmented brightness analysis as described in the manuscript provides brightness values that are immune to photodepletion.
