# Supplementary material for: Quantitative Measurement of Brightness from Living Cells in the Presence of Photodepletion
Source: PLoS One. 2014 May 12;9(5):e97440. doi: 10.1371/journal.pone.0097440 (PMC4018325; doi:10.1371/journal.pone.0097440)
Supplement: Text S3 — Fluorescence lifetime measurement. (DOCX) [file pone.0097440.s004.docx]

**Supporting Text S3**

**Fluorescence Lifetime Measurement**

Fluorescence lifetime measurements were performed on a two-photon microscope. Data were collected using the same experimental conditions as employed in FFS measurements except that the emission light passed through a polarizer set to magic angle conditions. The emission light was detected by a hybrid PMT (HPM-100-40, Becker & Hickl, Berlin, Germany) connected to time-correlated single photon counting module (TimeHarp 200, PicoQuant, Berlin, Germany). The reference timing signal of the TimeHarp module was obtained from the 80MHz clock output of the laser (Mai-Tai, Spectra Physics, Mountain View, CA) and subsequently filtered and amplified (91018 pulse conditioner, ISS Inc., Champaign, IL). The instrument response function was determined by recording second harmonic generation light obtained from urea crystals (ICN Biomedical, Aurora, OH). Experiments were performed on U2OS cells expressing EGFP. After measuring a fluorescence decay histogram in the absence of photodepletion ($f_{D}=0$) the cell was photobleached using the same protocol used for brightness experiments. A second measurement of the fluorescence decay histogram was performed on the photobleached cell. The fluorescence decay histograms were analyzed using routines written in IDL 8.0 (Research Systems, Boulder, CO).
